# Comparison of the characteristics of suicide attempters with major depressive disorder and those with no psychiatric diagnosis in emergency departments of general hospitals in China

**DOI:** 10.1186/s12991-017-0167-x

**Published:** 2017-12-01

**Authors:** Shengnan Wei, Haiyan Li, Jinglin Hou, Wei Chen, Xu Chen, Xiaoxia Qin

**Affiliations:** 1 Brain Function Research Section, First Affiliated Hospital, China Medical University, 155 Nanjing Road North, Shenyang, 110001 Liaoning People’s Republic of China; 2 Department of Radiology, First Affiliated Hospital, China Medical University, 155 Nanjing Road North, Shenyang, 110001 Liaoning People’s Republic of China; 3 Department of Psychiatry, First Affiliated Hospital, China Medical University, 155 Nanjing Road North, Shenyang, 110001 Liaoning People’s Republic of China

**Keywords:** Major depressive disorder, Attempted suicide, General hospital, Emergency department

## Abstract

**Background:**

Major depressive disorder (MDD) is a known major risk factor for suicide due to the high suicide mortality. However, studies comparing the characteristics of suicide attempters with major depressive disorder and those with no psychiatric diagnosis in China are very limited. This study examined and compared the sociodemographic and psychological characteristics of suicide attempters with MDD and those with no psychiatric diagnosis in emergency departments of general hospitals to better understand the risk factors for suicide attempts in China.

**Methods:**

All subjects were enrolled in the study between June 2007 and January 2008. A total of 127 suicide attempters—54 with MDD and 73 with no psychiatric diagnosis—were enrolled. The sociodemographic and clinical characteristics were compared between two groups using the statistical analysis performed using frequency distribution, Student’s *t* test, Chi-square test, and Fisher’s exact test and a logistic regression model.

**Results:**

Suicide attempters with MDD were more likely to be more depressive, older, divorced or separated, unemployed, and living alone, and more likely to write a suicide note, have suicide ideation, and be motivated by reducing pain and burden. Suicide attempters with no psychiatric diagnosis were more likely to be younger and more impulsive, have self-rescue, and be motivated by threatening or taking revenge on others. Multivariate logistic regression analysis identified the following independent predictors of suicide attempts in individuals with MDD: a lower score on the quality of life scale, more years of education, and suicide ideation.

**Conclusions:**

The present study found both similarities and differences in the sociodemographic and clinical characteristics of suicide attempters with MDD and those with no psychiatric diagnosis in the emergency departments of general hospitals in China. These findings will help us to recognize the characteristics of suicide attempters in both groups and develop specific interventions for the two types of suicide attempters to prevent future suicide in China. For example, the suicide attempters with MDD in the emergency departments must be advised to the psychological clinic.

## Background

Suicide is an important health problem worldwide and a leading cause of death worldwide, with over a million deaths per year [1, 2]. In China, suicide accounted for 3.6% of all deaths and was the fifth most common cause of death; we estimated a mean annual suicide rate of 23 per 100,000 and a total of 287,000 suicide deaths per year [3]. Suicide attempts are the single strongest predictor of suicide, because suicide attempts are even more frequent than completed suicide, occurring almost 20 times as often, and they are one of the strongest risk factors for completed suicide [4–6]. There are approximately 2 million suicide attempts per year in China. The study in Southeastern Turkey reported that most of these attempts appeared to be severe hopelessness with depression as a risk factor [7].

Major depressive disorder (MDD) is one of the most common mental disorders [8], with a lifetime incidence of 17.1% [9]. Around 15% patients with MDD ultimately die by suicide [10, 11]. MDD is a known major risk factor for suicide attempts due to the high prevalence of suicide attempts among people with MDD [12–14]. The study of the predictors of suicide attempts among patients with MDD showed that the risk of suicidal acts was almost exclusively confined to major depressive episode, with or without concurrent active substance abuse [15]. Meanwhile, one review also suggested that cannabis use was a relevant risk factor associated with both suicidal attempts and behaviors in psychotic and nonpsychotic samples [16]. Further, previous studies have shown that suicide attempters with MDD have their own socioeconomic and clinical characteristics [17–21]. For example, feelings of worthlessness are the single significant indicator of elevated risk of suicide attempt during a major depressive episode [21]. Suicide attempters with no psychiatric diagnosis also have their own epidemiological characteristics with respect to suicide [22]. Our previous study indicated that suicide attempters with mental illnesses are distinct in a number of important ways from those with no mental illness diagnosis. However, studies comparing the characteristics of suicide attempters with major depressive disorder and those with no psychiatric diagnosis in China are very limited.

We are not aware of any directly comparable characteristics between suicide attempters with major depressive disorder and those with no psychiatric diagnosis. Additionally, emergency departments are being increasingly recognized as an important setting for introducing suicide prevention measures, and studies have focused on developing effective interventions to initiate during emergency department stays for patients who have attempted suicide [23, 24]. To better understand the risk factors for suicide attempts in China and to develop a suicide prevention plan specific to this country, this study examined and compared the sociodemographic and psychological characteristics of two groups of suicide attempters who were treated in the emergency departments of general hospitals in China.

## Methods

### Design and setting

The individuals enrolled in this study were patients treated for suicide attempts (reported by the patient or family members) in the emergency departments of four tertiary-level general hospitals in Shenyang (population 6.9 million), Liaoning Province, in northeastern China. The hospitals were randomly selected from all tertiary general hospitals located in Shenyang using a random number table. All individuals who came to the emergency departments after making a suicide attempt were identified and approached by a trained research assistant.

Subjects were enrolled if they were 15 years of age or older, able to understand the study procedures, named at least one contact person (to enable follow-up), and provided written informed consent. The study was approved by the Institutional Review Board of China Medical University.

### Sample

Subjects were enrolled in the study between June 2007 and January 2008. A total of 239 suicide attempters completed the full evaluation; 127 (53.14%) individuals met the criteria for inclusion in the study, of whom 54 met the DSM-IV criteria for MDD and 73 had no documented DSM-IV Axis I disorders. There were no statistical differences in gender (*P* = 0.72), age (*P* = 0.89), or years of education (*P* = 0.51) between the 127 included and 112 excluded patients.

### Assessment

The 127 individuals and their accompanying family members were independently interviewed by two trained researchers. The comprehensive suicide attempt interview schedule that was used included several components that took a total of 2 h to complete as follows: A detailed structured questionnaire assesses the patients’ sociodemographic characteristics (age, gender, employment status, marital status, residence, annual family income, educational level, and religious beliefs) and the characteristics of the suicide attempt (method of self-harm, alcohol use at the time of the episode, reported motive, length of time suicide was considered before acting, presence of a suicide note, and help sought prior to attempt) along with self-reports of prior attempts and suicidal history among family members or associates. The Beck 19-item Scale for Suicide Ideation [25] evaluates the intensity of patients’ attitudes, behaviors, and plans to commit suicide. Each item consists of three options graded on a scale from 0 to 2 according to the intensity of the suicidality: the higher the score, the stronger the suicide ideation. The 24-item Hamilton Depression Rating Scale (HAMD) [26, 27] evaluates the depressive symptoms. A quality of life rating scale covering the month prior to the attempt evaluates six characteristics of the attempter (physical health, psychological health, economic circumstances, work, family relationships, and relationships with no family associates) on a scale from 1 (very poor) to 5 (excellent). Psychiatric diagnosis was made according to the Diagnostic and Statistical Manual of Mental Disorders, fourth edition, as assessed by the Structured Clinical Interview for DSM-IV Axis I Disorders (SCID). The Chinese translation of the SCID has been shown to be reliable and valid. This version of the SCID allows for the inclusion of “not otherwise specified” (NOS) categories of illness for subjects who have clinically significant symptoms combined with social dysfunction but do not meet the full criteria of a specific disorder (which is fairly common in China), and for the recording of multiple diagnoses ranked according to clinical importance. The six psychiatric researchers who participated in the study attended a 4-week training course on the use of the SCID; their interrater reliability at the end of training, tested using 16 taped interviews with different types of patients, was excellent (intraclass correlation coefficient = 0.95).

### Data analysis

The sociodemographic and clinical characteristics of suicide attempters with MDD and those with no psychiatric diagnosis were compared. To examine the mean differences in the study variables (i.e., age, gender, employment status, marital status, residence, self-reported history of self-harm, and family history of suicide), we used frequency distribution, Student’s *t* test, Chi-square test, and Fisher’s exact test with the assistance of the computer statistical package SPSS version 16.0 for Windows. The critical level of statistical significance was set at 0.05, and the analysis was two-tailed. A logistic regression model examined the factors associated with an MDD diagnosis among all suicide attempters, with MDD diagnosis as the dependent variable. Independent variables in the model included age, gender, race, marital status, employment status, living situation, years of education, religious beliefs, previous suicide attempts, self-rescue, motive for suicide attempt, presence of a suicide note, impulsive suicide attempts, suicide ideation, and quality of life. First, we entered all 15 variables into unconditional logistic regression analyses. The significant independent predictors from the analyses were then selected for possible use in the model. We tested forward (conditional) inclusion of variables in the logistic regression equation. Our model included 15 predictors and was based on complete data from 127 suicide attempters. Statistical significance in the logistic regression model was assessed using the Wald test, and the 95% confidence intervals were computed using the Gaussian approximation to the log likelihood of the rate.

## Results

### Sociodemographic characteristics of suicide attempters with MDD and suicide attempters with no psychiatric diagnosis

Of the 127 participants, 110 (86.61%) were female and 17 (13.39%) were male. A total of 54 participants met the DSM-IV criteria for MDD and 73 participants did not meet the criteria for any DSM-IV Axis I disorders. A comparison of demographic characteristics for each group is shown in Table 1. The average HAMD score was greater than eight points in the MDD group, and the average HAMD score was higher in the MDD group than in the group with no psychiatric diagnosis (*P* = 0.000). There were no significant differences in gender, race, annual family income, educational level, or religious beliefs between the groups. However, compared to suicide attempters with no psychiatric diagnosis, suicide attempters with MDD differed significantly in age, marital status, employment status, and living situation. Compared to attempters with no psychiatric diagnosis, suicide attempters with MDD were more likely to be older (*P* = 0.02), divorced or separated (*P* = 0.01), unemployed (*P* = 0.004), and living alone (*P* = 0.01), as well as less likely to be students (*P* = 0.004) or to live in shared accommodations (*P* = 0.01).

**Table 1:** Sociodemographic characteristics of suicide attempters with MDD and with no psychiatric diagnosis

	MDD (*n* = 54)		No psychiatric diagnosis (*n* = 73)		*T*	*P*
	Mean	SD	Mean	SD		
Age, years	36.39	15.39	29.88	14.29	2.46	0.02
Score on HAMD	27.70	7.78	6.56	5.49	17.07	0.000

	*N*	%	*N*	%	*χ* ^*2*^	*P*
Gender					0.04	0.83
Men	11	20.40	16	21.90		
Women	43	79.60	57	78.10		
Race					3.35	0.07
Han	47	87.00	70	95.90		
Minority	7	13.00	3	4.10		
Marital status					13.61	0.01
Never married	18	33.30	29	39.70		
Married	24	44.40	33	45.20		
Cohabitation	3	5.60	10	3.70		
Divorced/separated	4	7.40	0	0		
Widowed	5	9.30	1	1.40		
Employment status					13.53	0.004
Employment	30	55.60	46	63.00		
Unemployment	20	37.00	12	16.40		
Student	1	1.90	12	16.40		
Housewife	3	5.60	3	4.10		
Living situation					12.89	0.01
Solitary	9	16.70	1	1.40		
Shared accommodation	3	5.60	11	15.10		
Living with family	38	70.40	51	69.90		
Cohabitation	4	7.40	10	13.70		
Annual income of family						
Low (≤ 10,000)	13	24.10	10	13.70	2.43	0.30
Medium (10,001–50,000)	27	50.00	44	60.30		
High (> 50,000)	14	25.90	19	26.0		
Educational level					6.17	0.10
Illiterate	4	7.40	7	9.60		
Elementary school	24	44.40	38	52.10		
High school	13	24.10	22	30.10		
College	13	24.10	6	8.20		
Religious beliefs					0.71	0.40
No	48	88.90	68	93.20		
Yes	6	11.10	5	6.80		

*HAMD* Hamilton Depression Rating Scale

### Clinical characteristics of suicide attempters with MDD and suicide attempters with no psychiatric diagnosis

A comparison of the clinical characteristics of the two groups is presented in Table 2. The most common method of attempted suicide was self-poisoning for both the MDD and no psychiatric diagnosis groups (92.60 and 91.80%, respectively), and the majority of patients who overdosed used psychotropic drugs. No significant difference in the method of suicide attempt was observed between the two groups. There were also no significant differences in history of previous suicide attempts, family history of suicide attempts, alcohol use at the time of the episode or up to 12 h before, making of funeral arrangements, and seeking help. The suicide attempters with no psychiatric diagnosis had significantly more self-rescue and were more impulsive than the suicide attempters with MDD (*P* = 0.02, *P* = 0.000). The suicide attempters with MDD were more likely to write a suicide note and have suicide ideation than the suicide attempters with no psychiatric diagnosis (*P* = 0.02, *P* = 0.000). In terms of motive, the suicide attempters with MDD were more likely to want to reduce pain and burdens than the suicide attempters with no psychiatric diagnosis, and the suicide attempters with no any psychiatric diagnosis were more likely to want to threaten or take revenge on others than the suicide attempters with MDD (*P* = 0.000). The quality of life score was higher in the no psychiatric diagnosis group than in the MDD group (*P* = 0.000).

**Table 2:** Clinical characteristics of suicide attempters with MDD and with no psychiatric diagnosis

Characteristics	MDD (*n* = 54)		No psychiatric diagnosis (*n* = 73)		*χ* ^*2*^	*df*	*P*
	*N*	%	*N*	%			
Method of suicide attempts					0.76	2	0.69
Self-poisoning	50	92.60	67	91.80			
Self-injury	4	7.40	5	6.80			
Other	0	0	1	1.40			
Previous episodes of suicide attempts					0.75	1	0.39
No	41	75.90	60	82.20			
Yes	13	24.10	13	17.80			
Family history of suicide attempts					0.73	1	0.39
No	52	96.30	72	98.60			
Yes	1	3.70	1	1.40			
Alcohol use at the time of the episode or up to 12 h before					0.004	1	0.95
No	49	90.70	66	90.40			
Yes	5	9.30	7	9.60			
Self-rescue					7.19	1	0.01
No	53	98.10	61	83.60			
Yes	1	1.90	12	16.40			
Motive of suicide attempt					21.79	2	0.000
Deceased pain and burden	40	74.10	24	32.90			
Threatened or revenged others	9	16.70	38	52.10			
Other	5	9.30	11	15.10			
Suicide note					5.15	1	0.02
No	43	79.60	68	93.20			
Yes	11	20.40	5	6.80			
Funeral arrangements					1.50	1	0.22
No	50	92.60	71	97.30			
Yes	4	7.40	2	2.70			
Seeking for help					2.16	1	0.14
No	47	87.00	56	76.70			
Yes	7	13.00	17	23.30			
Impulsive suicide attempts					28.90	1	0.000
No	43	79.60	23	31.50			
Yes	11	20.40	50	68.50			
Suicide ideation					48.23	1	0.000
No	2	3.70	47	64.40			
Yes	52	96.30	26	35.60			
Score on quality of life (Mean, SD)	39.58	10.07	58.22	11.28	− 9.63		0.000

### Result of logistic regression analysis

A logistic regression model was used to examine the factors associated with an MDD diagnosis among all suicide attempters, with the presence of an MDD diagnosis as the dependent variable (Table 3). Multivariate logistic regression analysis identified the following independent predictors of having MDD in the suicide attempters: a lower score on the quality of life scale (OR = 0.85, 95% CI = 0.79–0.91), more years of education (OR = 1.30, 95% CI = 1.07–1.57), and suicide ideation (OR = 53.11, 95% CI = 5.87–480.45).

**Table 3:** Results of logistic regression analysis with 54 suicide attempters with MDD and 73 suicide attempters with no psychiatric diagnosis as dependent variables

Independents	*B*	SE	*P*	OR	95% CI
Continuous variables					
Score on quality of life	− 0.17	0.04	0.000	0.85	0.79–0.91
Years of education	0.26	0.10	0.01	1.30	1.07–1.57
Dichotomous variables					
Suicide ideation	3.97	1.07	0.001	53.11	5.87–480.45

## Discussion

### Main findings

In the present study, we found some similar sociodemographic and clinical characteristics between suicide attempters with MDD and those with no psychiatric diagnosis. For example, there were no significant differences between the two groups in gender, race, annual family income, education level, religious beliefs, method of attempted suicide, previous suicide attempt history, family history of suicide attempts, and making of funeral arrangements.

However, we also found some different sociodemographic characteristics between the two groups. First of all, the average HAMD score in the MDD group was greater than eight points, while the average HAMD score in the no psychiatric diagnosis group was less than eight points. This finding suggested that patients with MDD were more likely to attempt suicide during depressive episodes or when experiencing severe depressive symptoms. This result is consistent with the findings obtained in other studies [17, 19, 28, 29]. For example, depression severity is a predictor of vulnerability to suicide in patients with MDD [17], and suicide attempts among patients with MDD are strongly associated with the presence and severity of depressive symptoms [19]. Additionally, our results indicated that suicide attempters with no psychiatric diagnosis were younger and more likely to be students, and 47% (34/73) of suicide attempters with no psychiatric diagnosis were 24 years old or younger in this study. This finding is similar to those of the Chinese psychological autopsy study [30] and the European associated factors of attempted suicide study [31]. This evidence suggested that young people, who are more prone to suicide behavior, tend not to have a psychiatric diagnosis, whereas older people may attempt suicide due to mental disorders [32]. Indeed, the suicide attempters with MDD were older in our study, which is consistent with a recent finding that older residents with mental illness and depression seem to have a higher risk of suicide with high intent [33]. The age differences were similar to those found in Sandeep Grover et al. [22]; however, this finding was different from the findings of Steele et al. These inconsistent results may be due to cultural differences. In terms of marital and employment status, the suicide attempters with MDD were more likely to be divorced or separated and to be unemployed than those with no psychiatric diagnosis. This is consistent with previous findings that female MDD patients without any suicidality (no suicidal ideation, suicide plan, or history of attempted suicide) were significantly more likely to be married and employed [28], that employed status protected against high suicidality in patients with MDD [34], and that a lack of partner (marital status) was strongly associated with suicide attempts in MDD [19]. In terms of living situation, the suicide attempters with MDD were more likely to be living alone than those with no psychiatric diagnosis, likely because they were divorced or separated.

There were also some differences in the clinical characteristics of suicide attempters between the two groups. First, in terms of motive, the suicide attempters with MDD were more likely to be motivated by reducing pain and burden than the suicide attempters with no psychiatric diagnosis, and the suicide attempters with no psychiatric diagnosis were more likely to be motivated by threatening or taking revenge on others than the suicide attempters with MDD. This finding suggested that the suicide attempters with MDD had a greater desire to die, while those with no psychiatric diagnosis may not have truly wished to die. Patients with mental illnesses were more likely to die [35]. Some previous studies supported the view that mental illness, especially major depressive disorder [36–38], is an independent risk factor for suicide with high suicide intent [33, 39, 40] and found that self-harm of MDD patients was more motivated by depressive symptoms than self-harm in patients with no psychiatric diagnosis [22]. Second, we found that the suicide attempters with no psychiatric diagnosis had significantly more self-rescue and were more impulsive than the suicide attempters with MDD. This difference is easily explained given the different motives for suicide attempts in two groups. The suicide attempters with no psychiatric diagnosis had a lower expectation of actually dying, so they wanted to be helped when they attempted suicide, and they were more impulsive because of their motive to threaten or take revenge on others. This is consistent with other studies [22]. On the contrary, the suicide attempters with MDD were more likely to commit suicide to bring about death. Third, our results also demonstrated that the suicide attempters with MDD were more likely to write a suicide note and have suicide ideation than the suicide attempters with no psychiatric diagnosis. A previous study reported a similar finding that suicidal ideation was the most significant predictor of suicide attempt in depressed participants [20]. The reason for this may be explained by the finding that depression was related to a significantly increased risk of suicide attempted with high suicide intent [33]; previous studies have also found that suicide attempts with high intent were associated with higher lethality [41, 42]. Hence, suicide attempters with MDD were willing to write suicide notes. In addition, one study that examined impulsivity and suicide ideation reported that suicide attempts with low intent were associated with impulsivity [39], which is consistent with our findings. Finally, in our study, we found that the quality of life of the MDD group was lower than that of the no psychiatric diagnosis group. This is consistent with previous findings that stressful life events in people with MDD were significantly related to suicide attempts [28] and that people with MDD who had been exposed to certain stressful life events were at an elevated risk for future suicide attempts [18].

In summary, suicide attempters with MDD and with no psychiatric diagnosis shared some similar characteristics. However, suicide attempters with MDD were more likely to be depressed, older, divorced or separated, unemployed, and living alone, and to have lower quality of life, write a suicide note, have suicide ideation, and be motivated by reducing pain and burden. Meanwhile, suicide attempters with no psychiatric diagnosis were more likely to be younger and more impulsive, to have self-rescue, and to be motivated by threatening or taking revenge on others.

We also found that the predictors of suicide attempters with MDD in emergency departments were lower quality of life, higher education level, and suicide ideation. These results suggest that suicide attempts in people with lower quality of life and suicide ideation may be related to MDD. This is consistent with previous studies [18, 20, 28]. Higher education level was the key factor associated with MDD, which suggests that the number of years of education is the most meaningful predictor of suicide attempts in patients with MDD. The reason for this may be due to the age differences of the MDD and no psychiatric diagnosis groups in our sample, or due to the sample size. And it requires further study to be conducted in China.

### Strengths

This study has several strengths. For one, it is one of the few studies that have focused on the detailed assessment and comparison of suicide attempters with MDD and those with no psychiatric diagnosis treated in the emergency departments of general hospitals in China. The samples were representative of emergency outpatients at general hospitals in a large urban municipality in northern China. The assessment of suicide attempters was based on a clinical structured interview and standardized instruments, rather than on a psychological autopsy study. The diagnosis was based on the administration of SCID by trained researchers who had excellent interrater reliability, and rigorous quality control measures were enforced throughout the study. The healthcare delivery system in Shenyang is similar to that in other urban areas of China; thus, we believe that the results can be extrapolated to other urban areas in China. There are, however, several limitations that should be considered when interpreting the results.

### Limitations

Several limitations of the present study should be acknowledged. First, the assessments of Axis I disorders according to the DSM-IV in our study were performed, not the Axis II diagnosis. Second, we had limited data on suicide attempters who did not participate in our study. Third, the findings are based on relatively small sample numbers from regional hospitals, raising the question of whether the results can be generalized. Finally, our study cohort was limited to an urban population only, and different outcomes may be expected in a rural population. Thus, a multicenter study focusing on multiple sites is required to demonstrate current trends in attempted suicide in China. Despite these limitations, the present study provided evidence that will help researchers and clinicians understand the characteristics of suicide attempters with MDD and those with no psychiatric diagnosis in China, and develop interventions to prevent future suicide.

## Conclusions

The present study found both similarities and differences in the sociodemographic and clinical characteristics of suicide attempters with MDD and those with no psychiatric diagnosis in the emergency departments of general hospitals in China. These findings will help us to recognize the characteristics of suicide attempters in both groups and develop specific interventions for the two types of suicide attempters to prevent future suicide in China. For example, the suicide attempters with MDD need to actively go to the psychological clinic, especially medication. By contrast, the suicide attempters with no psychiatric diagnosis need more mental health education to understand how to deal with stress and crisis.

## Abbreviations

MDD: major depressive disorder
HAMD: the Hamilton Depression Rating Scale
SCID: the Structured Clinical Interview for DSM-IV Axis I Disorders
